# Precision and personalized medicine: What their current definition says and silences about the model of health they promote. Implication for the development of personalized health

**DOI:** 10.3389/fsoc.2023.1112159

**Published:** 2023-02-21

**Authors:** Cyrille Delpierre, Thomas Lefèvre

**Affiliations:** ^1^Centre for Epidemiology and Research in POPulation Health (CERPOP) UMR1295, INSERM-Université Toulouse III, Toulouse, France; ^2^Institut de Recherche Interdisciplinaire sur les Enjeux Sociaux (IRIS) CNRS UMR8156 INSERM U997 EHESS USPN, Paris, France

**Keywords:** precision medicine, personalized health, health model, challenges, risks

## Abstract

The US National Human Genome Research Institute defines precision medicine as follows: “Precision medicine (generally considered analogous to personalized medicine or individualized medicine) is an innovative approach that uses information about an individual's genomic, environmental, and lifestyle information to guide decisions related to their medical management. The goal of precision medicine is to provide a more precise approach for the prevention, diagnosis, and treatment of disease.” In this perspective article, we question this definition of precision medicine and the risks linked to its current practice and development. We highlight that in practice, precision medicine is based on the use of large volumes of biological data for individual purposes mostly in line with the biomedical model of health, which carries the risk of the biological reductionism of the person. A more comprehensive, precise, and even “personal” approach to health would require taking into account environmental, socio-economic, psychological, and biological determinants, an approach more in line with the biopsychosocial model of health. The role of environmental exposures, in a broad sense, is highlighted more and more, notably in the field of exposome research. Not considering the conceptual framework in which precision medicine is deployed leads to the concealment of the different responsibilities that can be mobilized within the health system. Anchoring precision medicine in a model that does not limit its definition to its biological and technical components makes it possible to envisage a personalized and more precise medicine, integrating a greater share of interventions centered on the skills and life contexts of individuals.

## Some elements of definition

The last 20 years have seen the emergence of the concepts of P4 medicine, that is, participatory, personalized, predictive, and preventive medicine (Hood and Friend, 2011), and this has occurred mainly in parallel with the developments in clinical genetics, artificial intelligence, and digital technology. P4 medicine reflects fairly well the consideration of developments mainly driven by technology rather than theory. P4 medicine is particularly based on the now almost ubiquitous nature of digital technology, whether in terms of data collection (e.g., *via* internet browsing, or simply the use of smartphones), storage, and computing capacities (especially for data genome sequencing in clinical routines), or in terms of the development and use of algorithms. It updates an old idea and desire: that of gaining knowledge and information that is more specific, more targeted, and more adapted to the pathology of a given person, and to be able to analyze these data in an intelligible and useful form in order to be able to propose personalized treatment and care.

The concept of P4 medicine is thus mainly based on the use of large volumes of data, mostly biological (omics data). This is particularly the case for personalized medicine, which is one of the foundations of P4 medicine. The US National Human Genome Research Institute defines personalized medicine as “an emerging practice of medicine that uses an individual's genetic profile to guide decisions made in regard to the prevention, diagnosis, and treatment of disease. Knowledge of a patient's genetic profile can help doctors select the proper medication or therapy and administer it using the proper dose or regimen” (National Human Genome Research Institute, n.d.). It is specified that “personalized medicine is being advanced through data from the Human Genome Project,” highlighting the crucial importance of genetics to this approach.

This definition is similar to the definition given by the US National Cancer Institute, which defines personalized medicine as “a form of medicine that uses information about a person's own genes or proteins to prevent, diagnose, or treat disease. In cancer, personalized medicine uses specific information about a person's tumor to help make a diagnosis, plan treatment, find out how well treatment is working, or make a prognosis.” It is interesting to note that the US National Cancer Institute uses the same definition for precision medicine (National Cancer Institute, n.d.).

In practice, precision medicine and personalized medicine are often used interchangeably. This is recognized by the US National Human Genome Research Institute in its definition of precision medicine: “Precision medicine (generally considered analogous to personalized medicine or individualized medicine) is an innovative approach that uses information about an individual's genomic, environmental, and lifestyle information to guide decisions related to their medical management. The goal of precision medicine is to provide more a precise approach for the prevention, diagnosis, and treatment of disease” (National Human Genome Research Institute, n.d.).

According to the US National Research Council, “personalized medicine is an older term with a meaning similar to precision medicine. However, there was concern that the word “personalized” could be misinterpreted to imply that treatments and preventions are being developed uniquely for each individual. The Council therefore preferred the term precision medicine to personalized medicine” (National Library of Medicine, 2019).

We believe that these two terms are not interchangeable, as personalized medicine incorporates broader dimensions than those explored in practice in precision medicine as explained below.

## What is done in practice when we talk about precision and personalized medicine?

In practice, the notion of precision medicine remains centered on the use of large volumes of data, in terms of the people included but also in terms of the data used, the latter being largely biological (for example, genomics, transcriptomics, epigenomics, proteomics, metabolomics, and pharmacogenomics) and for individual-centered purposes and applications. In fact, it is largely intended for predictive medicine, for the determination of risks calculated from groups of individuals with the same biological and clinical characteristics, or for diagnostic decisions, by multiplying individual biological data, for “à la carte” health. The overall aim is to offer patients a treatment adapted to their biological and clinical characteristics. Precision medicine consists of identifying which approaches/treatment will be effective for which patients according to the group to which they belong on the basis of their biological characteristics. In this sense, it is more stratified medicine than personalized medicine. As an illustration of what precision medicine means in routine practice, we can refer to oncology, where there has been significant developments in this type of medicine. The French National Cancer Institute states that precision medicine is currently based on two types of treatment, targeted therapies and specific immunotherapy (144 drugs available, including 107 targeted therapies and 37 specific immunotherapies) (French National Cancer Institute, n.d.). These treatments are mainly used for patients with advanced forms of cancer or who have relapsed. It is thus used to offer patients a treatment adapted to the characteristics of their tumor, and has had success in cases including chronic myeloid leukemia, lung and breast cancer, and metastatic melanoma (Gambardella et al., 2020).

## Precision and personalized medicine: A definition that implicitly reflects one of the two main models of health

Historically, there are two main models of health representation: the biomedical or biological model (Yuill et al., 2010) and the biopsychosocial model proposed in the 1970s by Engel (1977). The biomedical model intends to define health on the basis of the individual's health, this health itself being defined and determined by the biology of the individual at different scales: for example, genetics at the most basic scale today, and the molecular, cellular, histological, and anatomical scales. This representation centered on the individual is supplemented with the notion of the environment, to take into account everything that is not the individual. To refer to health as a whole, we then speak in a very broad and not very explicit definition of a gene × environment interaction model. It is therefore initially a deeply reductionist and materialist representation. This model of health is the one on which precision medicine as described above is built, which is largely aimed at characterizing individuals by their biological characteristics. Conversely, the biopsychosocial model defines individual health by taking into account at least three complementary dimensions: the biological, psychological, and social dimensions. This definition is in accordance with the WHO definition of health as a “state of complete physical, mental, and social wellbeing, not merely the absence of disease or infirmity” (World Health Organization, 2013). This definition of health is then not entirely covered by that of pathology, and therefore covered even less by the reduction to the biological determination of a pathological risk. It also proposes to take into account environmental, socio-economic, and psychological determinants in addition to the biological dimension, and makes health a complex, interdisciplinary, and transdimensional field. By proposing to integrate other dimensions than the biological, this model offers a more global approach to health that is more comprehensive, more precise, and even more personal since it considers a person in multiple dimensions. The biopsychosocial model is therefore more in line with a medicine that could be defined as personalized.

However, the practice of precision medicine, maintaining health essentially as a matter of disease and biological reductionism, can distract from the need to consider the social and environmental determinants of health (Mentis et al., 2018). This model is all the more relevant as it is becoming increasingly clear that taking into account omics characteristics (primarily genomic ones) alone is not sufficient to perfectly predict the phenotype, such as the risk of developing a disease or the response to a treatment, and that the role of environmental exposures (including physicochemical, behavioral, and psychosocial exposures) is fundamental in the way genes are expressed. According to the International Agency for Research on Cancer, 40% of cancers in France can be attributed to lifestyle or environmental factors (International Agency for Research on Cancer, n.d.). The development of the so-called exposome science highlights the rebalancing of the environmental gene balance in favor of the weight of the environment in a broad definition. The concept of the exposome refers to all the exposures to external and environmental factors that an individual undergoes during his or her life from the prenatal period. It includes the external exposome, which refers to exposures outside the body that may be general (e.g., social, cultural, and ecological contexts) or specific (e.g., chemical pollutants or lifestyle factors), and the internal exposome, which refers to measurable biomarkers and metabolic and physiologic processes inside the body (Wild, 2005, 2012). Interestingly, the internal exposome builds on fields of study such as genomics, transcriptomics, metabolomics, and lipidomics, which are at the core of precision medicine. The exposome approach thus provides a conceptual framework for linking the external environment (external to the organism), including the totality of human environmental exposures from conception onwards, to internal biological functioning (internal exposome). This relationship between the environment, in particular the social environment, and biological functioning has been further conceptualized and formalized in social epidemiology. The concept of embodiment that Nancy Krieger has developed refers to “the way in which we literally incorporate biologically the material and social world in which we live” (Krieger, 2005). The way in which this biological embodiment of the social, or the social to biological transition (Blane and Kelly-Irving, 2013; Kelly-Irving and Delpierre, 2021), can take place through two broad main types of socially distributed initial mechanisms that can interact and affect each other along the life course. Firstly, mechanisms of “exogenous” origin, though which entities or conditions external to the body either enter the body and elicit a physiological response from it (for example, inert or living entities like foodstuffs, asbestos, viruses, bacteria, and pollutants) or lead to physical harm (e.g., injuries and accidents) or exertion (e.g., movements and actions). This concerns environmental exposures such as pollution, pesticides, work exposures, and lifestyle behaviors such as tobacco use, alcohol use, and diet. Secondly, mechanisms of “endogenous” origin, through which sensory interpretations of interactions with the environment elicit responses from “internal” molecules from the body mainly linked to stress perception and stress response systems as well as cognitive and psychological functions. In terms of exposures, these concern especially psychosocial exposures such adversities during childhood (for example, trauma, sexual abuse, physical violence, and neglect), occupational constraints, social support, social isolation, and experiencing discrimination, and whether they are related to age, gender, social class, skin color, sexual orientation, or disability (Blane and Kelly-Irving, 2013; Kelly-Irving and Delpierre, 2021). It is therefore scientifically inappropriate to consider genes and the social environment separately, as the biological functioning of an individual is closely linked to the environment in which he or she evolves.

## Why it is important to develop a personalized medicine and health based on a broader vision of health

There is considerable evidence of the influence of various external exposures on biological functioning at different omics levels in both animals and humans, including gene expression through epigenetic changes. Among the environmental exposures studied, a great deal of data is available on the effect of physicochemical exposures or health-related behaviors. The effect of the social environment is less frequently taken into account, as a review of the literature has just shown in research on the exposome, despite research calling for them to be taken into account (Senier et al., 2017; Vineis and Barouki, 2022). However, the effect of the social environment and psychosocial exposures on biological functioning has been highlighted in the literature, including at the omics level (Lang et al., 2020; Palma-Gudiel et al., 2020; Lim et al., 2022), underlining the interest of integrating this dimension to better understand biological functioning, health, and disease. Horton has talked about a syndemic rather than a pandemic when regarding the COVID-19 epidemic, highlighting how the joint consideration of social and biological aspects improves the understanding and management of the disease (Horton, 2020).

Ziegelstein proposed that a new “omics” term called “personomics” be added to the precision medicine toolkit (other omics approach) as the missing link in the evolution of precision medicine to a medicine that would be really personalized. He stipulated that “Personomics recognizes that individuals are not only distinguished by their biological variability, but also by their personalities, health beliefs, social support networks, financial resources, and other unique life circumstances that have important effects on how and when a given health condition will manifest in that individual and how it will respond to treatment” (Ziegelstein, 2017). This interesting proposal takes up the idea that a real “personalized medicine and health” needs to include information about the person's environment and living conditions and not only the biological characteristics of their disease. Such an approach presupposes the availability of environmental and socio-economic data which are very rarely present in medical records or simply not systematically searched for by physicians. While individuals are increasingly phenotyped at the “omics” level, they are rarely phenotyped at the environmental level (e.g., the physicochemical, behavioral, psychological, and social levels). Personalized medicine implies making the same effort to characterize the person in terms of his or her living conditions as was made to characterize the patient at the biological level.

## Specificities of current personalized medicine and implications in terms of means of action and care

### Technique as the main defining element of personalized medicine

Any practice is marked by the tools at its disposal, as they determine the possibilities of concrete actions, and the ways in which these actions are carried out. Thus, our conceptions of health, illness, and what can be prevented, treated, restored, or palliated are intimately linked to our knowledge, our societies, and our techniques. It is still quite rare that a technique emerges and is applied in the most appropriate and direct way for a previously identified health problem. This refers to the concept of situated knowledge, developed by Haraway (1988), which postulates that knowledge is embedded in, and therefore affected by, the concrete, historical, cultural, linguistic, and value context of the person or entity producing it.

However, it seems that the current definition of personalized medicine marks a turning point or at least an accentuation: that is, a significant imbalance between what technique determines practice and knowledge, and what knowledge and practice determine accepted and desirable techniques. Indeed, it appears that personalized medicine as it is presently is mainly defined by its technical aspects: the use of data, directly or *via* more or less sophisticated algorithms. Above all, it does not respond to a conceptual framework of health or healthcare. In this sense, it may seem to escape us, since we have not imposed any particular framework on it, at least explicitly. However, medicine, and more generally, health, do not have direct access to the modalities of existence and use of this technique, whose main infrastructures and proposed tools are in the hands of companies themselves unrelated to health, such as GAFAMS.

### The choice and explanation of the conceptual framework of health and care from which personalized medicine is defined and practiced determine the possible means of action

If we choose to anchor personalized medicine, i.e., its definition (what it does, how, and why) and its means of action, in the biomedical model, the implications in terms of the responsibility of the actors and the means of action will be essentially centered on the individual. In particular, the debate around individual responsibility, and therefore the financing of health and healthcare according to individual health risks, is making a comeback: data-driven techniques and tools are deemed to provide more resources, the purpose of which is to screen the risks and quantify them, more or less upstream. The means of action are and will remain mainly directed toward the individual and the biological levers. If we anchor personalized medicine in the biopsychosocial model, the implications are different, again in terms of responsibilities and means of action. The individual responsibilities and biological actions of the previous model are replaced or supplemented by shared responsibilities and funding mutualized or endorsed by actors other than the individual (particularly in the case of environmental risks linked to several types of pollution), and also by broader means of action. We have mentioned the possibility of taking into account the patient's environment and living conditions in the physician-patient relationship, but this goes even further, since we are also dealing with public health policies, as well as collective means of action. Moreover, we must consider that health crosses a large number of policies that are not specifically restricted to public health, e.g., industrial, agri-food, education, and housing policies. The prescription of diets and physical activity, for example, is part of this logic if we consider behavior. Interventions for stress management are also part of this logic. This opens up a range of actions and professionals who can intervene in individual health management. Inhibiting the conceptual framework in which personalized medicine is deployed means obscuring the various responsibilities that can be mobilized within the healthcare system, but also depriving ourselves of additional means of action, including more precise means: since 2015, there has been talk of “precision public health.” Finally, anchoring personalized medicine in a model that is not only biomedical but also does not restrict its definition to its technical components, makes it possible to envisage a personalized and more precise medicine—more relevant?—integrating a greater amount of interventions and human interrelations centered on personal skills and contexts (e.g., therapeutic education and patient empowerment). Of course, the alternative to the two existing models, the biomedical and biopsychosocial, may no longer be sufficient, and a new framework must be proposed.

## Data availability statement

The original contributions presented in the study are included in the article/supplementary material, further inquiries can be directed to the corresponding author.

## Author contributions

All authors listed have made a substantial, direct, and intellectual contribution to the work and approved it for publication.
